# Nerve transfer for restoration of lower motor neuron-lesioned bladder function. Part 2: correlation between histological changes and nerve evoked contractions

**DOI:** 10.1152/ajpregu.00300.2020

**Published:** 2021-03-24

**Authors:** Mary F. Barbe, Courtney L. Testa, Geneva E. Cruz, Nagat A. Frara, Ekta Tiwari, Lucas J. Hobson, Brian S. McIntyre, Danielle S. Porreca, Dania Giaddui, Alan S. Braverman, Emily P. Day, Mamta Amin, Justin M. Brown, Michael Mazzei, Michel A. Pontari, Ida J. Wagner, Michael R. Ruggieri

**Affiliations:** ^1^Department of Anatomy and Cell Biology, Lewis Katz School of Medicine, Temple University, Philadelphia, Pennsylvania; ^2^Department of Electrical and Computer Engineering, College of Engineering, Temple University, Philadelphia, Pennsylvania; ^3^Drexel University College of Medicine, Philadelphia, Pennsylvania; ^4^Department of Neurosurgery, Massachusetts General Hospital, Boston, Massachusetts; ^5^Department of Surgery, Lewis Katz School of Medicine, Philadelphia, Pennsylvania; ^6^Department of Urology, Lewis Katz School of Medicine, Temple University Health System, Philadelphia, Pennsylvania

**Keywords:** bladder, detrusor muscle, ex vivo bladder smooth muscle strips, histology, muscle contractility

## Abstract

We determined the effect of pelvic organ decentralization and reinnervation 1 yr later on urinary bladder histology and function. Nineteen canines underwent decentralization by bilateral transection of all coccygeal and sacral (S) spinal roots, dorsal roots of lumbar (L)7, and hypogastric nerves. After exclusions, eight were reinnervated 12 mo postdecentralization with obturator-to-pelvic and sciatic-to-pudendal nerve transfers, then euthanized 8-12 mo later. Four served as long-term decentralized only animals. Before euthanasia, pelvic or transferred nerves and L1–S3 spinal roots were stimulated and maximum detrusor pressure (MDP) recorded. Bladder specimens were collected for histological and ex vivo smooth muscle contractility studies. Both reinnervated and decentralized animals showed less or denuded urothelium, fewer intramural ganglia, and more inflammation and collagen, than controls, although percent muscle was maintained. In reinnervated animals, pgp9.5+ axon density was higher compared with decentralized animals. Ex vivo smooth muscle contractions in response to KCl correlated positively with submucosal inflammation, detrusor muscle thickness, and pgp9.5+ axon density. In vivo, reinnervated animals showed higher MDP after stimulation of L1–L6 roots compared with their transected L7–S3 roots, and reinnervated and decentralized animals showed lower MDP than controls after stimulation of nerves (due likely to fibrotic nerve encapsulation). MDP correlated negatively with detrusor collagen and inflammation, and positively with pgp9.5+ axon density and intramural ganglia numbers. These results demonstrate that bladder function can be improved by transfer of obturator nerves to pelvic nerves at 1 yr after decentralization, although the fibrosis and inflammation that developed were associated with decreased contractile function.

## INTRODUCTION

Incontinence following injury that interrupts the lower motor neurocircuitry controlling the bladder often has lasting consequences on one’s quality of life. This condition can be a source of anxiety and depression due to persistent dependence on others for personal care (1, 2). A 2012 systematic review found that individuals who sustain spinal cord injury prioritize recovery of bladder function above most other sustained deficits (3).

Current interventions for an atonic neurogenic bladder following a lower motor neuron lesion, such as indwelling or intermittent catheterization (4), fail to target the source of the pathology and increase patient susceptibility to urinary tract infections (5). With the goal of targeting recovery of bladder function following a lower motor neuron injury, our group developed a model of surgical reinnervation via transfer of nerves that originate from other, higher spinal cord segments to either the anterior vesicle branch of the pelvic nerve to restore bladder contraction or to the pudendal nerve to restore external urethral and anal sphincter contraction (6–14). In several studies, we used nerves originating from upper lumbar regions of the spinal cord, including obturator, femoral, and genitofemoral nerve branches, with success in reinnervation of the bladder immediately following, or 1, 3, or 12 mo after sacral decentralization. Specifically, immediate transfer of femoral or genitofemoral nerve branches to anterior vesicle branches of pelvic nerves resulted in strong nerve- or spinal root-evoked bladder contractions and urine flow in 29 of 36 animals (81%), when assessed at 4.5 to 12 mo postreinnervation (7, 13). Postdecentralization delays of 1 or 3 mo before similar transfer of genitofemoral nerves to anterior vesicle pelvic nerve branches, resulted in nerve-evoked bladder contractions and urine flow in three of four dogs and four of six dogs, respectively (70% when combined), when assessed at 7 mo postreinnervation (15). In a recent interim study, a postdecentralization delay of 12 mo, then transfer of obturator nerves to anterior vesicle pelvic branches, resulted in nerve-evoked bladder contractions and urine flow in two of three reinnervated dogs when assessed over 10 mo postreinnervation (14). One of the reinnervated dogs even showed clear evidence of voluntary voiding during awake bladder filling, due to either a functional bladder contraction or a voluntary Valsalva maneuver (14).

Surgical reinnervation of the bladder, as with any surgery, is not without risk. It is critical to determine the upper limit of the timeframe in which a successful reinnervation could be pursued following lower spinal root injury. Assessment of bladder integrity at 4 mo after decentralization, with or without an immediate postdecentralization genitofemoral nerve transfer as a reinnervation strategy, revealed healthy-looking intramural ganglia and large myelinated nerve bundles traversing the bladder wall and detrusor muscle layer in both decentralized and reinnervated animals compared with sham-operated control animals (16). In addition, assessment of bladder integrity at 12 mo postdecentralization also revealed healthy looking intramural ganglia, although less robust pelvic nerve-evoked bladder contractions than in sham-operated control animals (17). However, there was no sign of detrusor muscle layer atrophy, and bladder smooth muscle still showed contractility in ex vivo electric field stimulation studies (17), opening the possibility for successful reinnervation of the bladder after long-term decentralization.

Our main objective here was to extend these studies to now examine, for the first time, the effects of long-term decentralization of 9–13 mo and then reinnervation (followed by an 8–12 mo postreinnervation recovery period) versus the effects of 11–21 mo of decentralization on the histology of the urinary bladder compared with sham/unoperated control animals. Establishment of the canine model and interim pilot study results have been published previously and will be referred to here after as the pilot study of this series of investigations (14). Our second objectives were: *1*) to correlate the histological findings with functional in vivo bladder pressure findings evoked by electrical stimulation of intact pelvic nerves, transferred peripheral nerves, or spinal roots, and *2*) to correlate the histological findings and in vivo functional findings with ex vivo smooth muscle strip contractions in response to KCl, ATP, or electric field stimulation as an extension of Part 1 of this study (18).

## MATERIALS AND METHODS

### Animals

Studies were approved by the Temple University Institutional Animal Care and Use Committee, in accordance with the guidelines of the National Institute of Health for the Care and Use Laboratory Animals, United States Department of Agriculture (USDA), and Association for Assessment and Accreditation of Laboratory Animal Care (AAALAC). Twenty-six female mixed-breed hounds were used (6–8 mo of age, 20–25 kg, at experimental onset; Covance Research Products, Inc., Lancaster County, PA, or Marshall BioResources, North Rose, NY). The dogs were provided free access to food and water and maintained in a 12:12-h light-dark cycle. They were housed in large, connected dog pens, typically with two cage companions.

### Original Design and Necessary Modifications of the Project Plan

**Figure 1.**
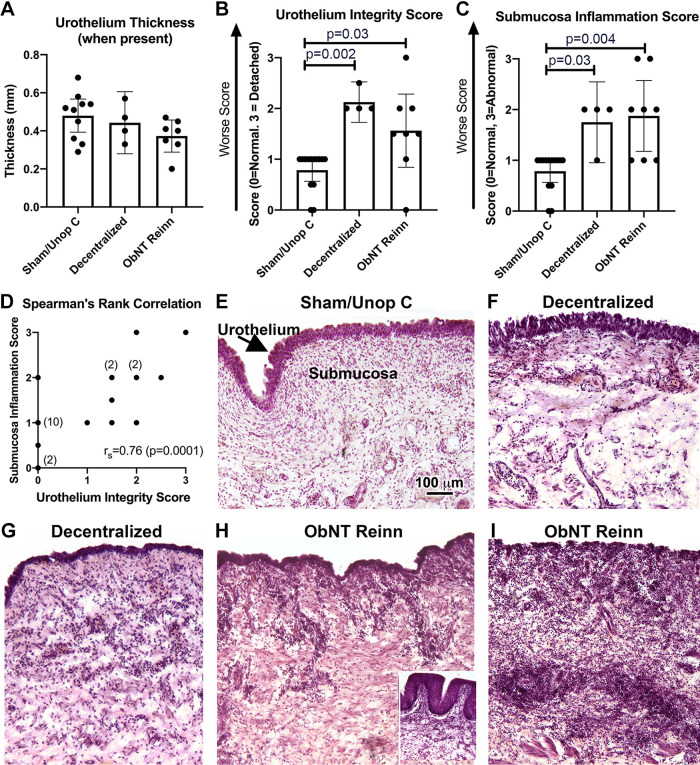
Urothelium and submucosa histological integrity. Hematoxylin and eosin (H&E) stained sections were used for these assays. *A*: urothelium thickness was assayed, when present. *B*: urothelium integrity score for which 0 = normal and 3 = detached. *C*: submucosa inflammation score in which 0 = normal and 3 = abnormal (very inflamed). Means and 95% CI are shown in panels *A*–*C*. *D*: Spearman’s rank correlation (*r*_s_) between submucosa inflammation and urothelium integrity scores. When ≥ 2 animals had the same score, the number per finding is indicated in parentheses. *E–I*: representative images for each group, with two images provided for the Decentralized group and three for the ObNT Reinn group (the 3rd in the *inset* in *H*) to show the observed variability. A Brown–Forsyth ANOVA was used for the urothelium thickness analysis and Kruskal–Wallis ANOVAs were used for the inflammatory score analyses. Scale bar in *E* is applicable to *F–I*. CI, confidence interval.

**Figure 2.**
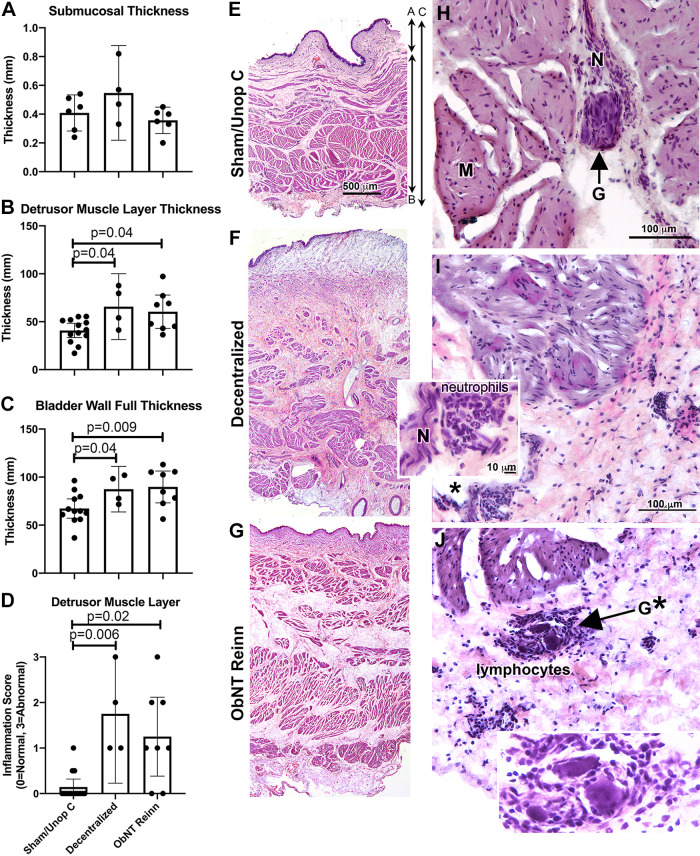
Thicknesses (height) of the submucosa, detrusor muscle layer, and full bladder wall (from urothelium to serosa), and detrusor muscle layer inflammatory response. *A*: submucosa thickness. *B*: detrusor muscle layer thickness. *C*: bladder wall full thickness. *D*: detrusor muscle layer inflammation score. Means and 95% CI are shown in panels *A–D*. *E–G*: low power representative images. Double arrows on the right side of *E* shows bladder wall zones measured for data shown in *A–C*. *H–J*: higher power images of the detrusor muscle layer. *Regions enlarged in *insets* of *I* and *J*. G, intramural ganglia; M, muscle; N, nerve. Brown–Forsyth ANOVAs were used for the layer analyses and a Kruskal–Wallis ANOVA was used for the inflammatory score analysis. Scale bar in *E* is applicable to *F* and *G*; scale bars in *H* and *I* are applicable to *J*. CI, confidence interval.

The original design and necessary modifications of the project plan are as described in Part 1 of this series (18). Accordingly, the current study reports results from 26 animals: *group 1*, eight functionally decentralized by transecting all spinal roots caudal to L7, hypogastric nerves, and L7 dorsal roots, for 9–13 mo (followed by a 12-mo recovery), then reinnervated and followed for an additional 7- to 12-mo recovery (average of 10 mo post reinnervation recovery, ObNT Reinn animals); *group 2*, four animals that underwent similar decentralization followed by an 11- to 21-mo recovery (average of 18 mo, Decentralized); and *group 3*, 14 normal controls (11 sham-operated and 3 unoperated, Sham/Unop). Figures outlining the surgical procedures were provided in in Fig. 2 of the pilot study of this series (14) and as a flow chart in Fig. 1 of Part 1 of this series (18).

This is Part 2 of a series. There is some overlap with the pilot study (14) and Part 1 (18) of this series. Specifically, in vivo electrophysiology data from the pilot study was included for three of the ObNT Reinn, one of the Decentralized, and six of the sham-operated animals. The ex vivo muscle strip data presented in Part 1 (18) was correlated with the morphological data of this current study.

### Surgical Decentralization of the Bladder

Surgical decentralization procedures were as described in the pilot study (14) and Part 1 (18) of this series. Briefly, immediately before surgery, the dogs were sedated with propofol (6 mg/kg iv) to allow endotracheal tube insertion, and then anesthesia was maintained using isoflurane (2%–4% maximum alveolar concentration) with oxygen as the carrier gas. Catheterization was performed for placement of balloon catheters through the urethra and into the bladder (17). All animals except for the unoperated controls underwent a laminectomy of L6-S2 vertebrae to expose the lower spinal cord and spinal roots, and L7 and S1-3 ventral roots were identified electrophysiologically, using the previously described methods (7, 15). Decentralized and ObNT Reinn animals used in this study were decentralized by bilateral transection of the hypogastric nerves, bilateral extradural transection of dorsal roots of L7, and bilateral extradural transection of dorsal and ventral roots of S1–3. Hypogastric nerves were accessed via abdominal surgery and were bilaterally transected, with 10- to 15-mm length excised, from their proximal emergence from the inferior mesenteric ganglion to their distal entrance into the pelvic plexus. Sham-operated controls underwent lumbosacral laminectomy, nerve root identification via electrical stimulation without root transection, and abdominal laparotomy with identification of hypogastric nerves. As no differences were observed between the sham-operated and unoperated controls in this or prior studies (13, 17), these animals were combined into one sham/unoperated control group (Sham/Unop C).

### Nerve Transfer Surgeries at 12 Mo after Decentralization

For the nerve transfer surgery, eight animals were anesthetized and catheterized with balloon catheters. Obturator nerves were accessed via abdominal surgery, identified, and divided longitudinally using a microscalpel. Approximately 75% of the obturator nerve was left intact to retain innervation of hind limb adductor muscles. The other quarter of the fascicles were transected, transferred, and sutured end-to-end to the transected anterior vesical branch of the pelvic nerve, bilaterally, using previously described methods (13, 14). Axoguard nerve connectors (Axogen Corp, Alachua FL) were used to maintain transferred nerve coaptation and to reinforce the coaptation site that was covered in Tisseel fibrin sealant (Baxter, Deerfield, IL).

For functional reinnervation of the external urethral and anal sphincters, a redundant branch of the sciatic nerve was transferred to branches of the pudendal nerve that induced urethral and anal sphincter contractions with intraoperative electrical stimulation. The effects of the pudendal nerve transfer on the urethral and anal sphincters are Part of another study and are not presented in this manuscript, portions of which have been previously reported (14).

### Postoperative Care

Postoperative care procedures were as described in Part 1 of this series (18).

### Retrograde Dye Injection

Three weeks before euthanasia, the bladder wall was cystoscopically injected into four sites around the ureterovesical junction with Fluoro-Gold (4%) wt/vol in 0.9% saline, Fluorochrome, LLC, as described previously (17).

### In Vivo Functional Electrical Stimulation at 18 Mo Postdecentralization, or 10 Mo after Reinnervation Surgery

An average 10-mo reinnervation recovery time was chosen based on data from the pilot study of this series that showed functional recovery of squat and void postures between 4 and 6 mo after obturator nerve transfer in three ObNT Reinn animals (14). Before euthanasia, the animals were reanesthetized and catheterized, as described in *Surgical Decentralization of the Bladder*. Bladder pressures were monitored throughout the surgeries, as were vital signs. Right and left pelvic nerves in the Sham/Unop C and Decentralized animals or transferred nerves (obturator-to-pelvic) in ObNT Reinn animals were stimulated with either a monopolar or bipolar electrode using a current of 0.5–10 mAmp, a frequency of 20 Hz, and a pulse duration of 0.2 ms in a train of 4- 7-s duration. Changes in pressures were continuously recorded with external pressure transducers interfaced with the PowerLab multichannel data acquisition system and LabChart software (ADInstruments, Colorado Springs, CO). Strength of nerve-evoked bladder contractions after pelvic or proximal obturator nerve stimulation was derived from differences between the resting baseline pressure and the peak pressure obtained during continuous stimulation. The resulting maximum detrusor pressure (MDP) in cmH_2_O is reported.

In addition, L1–S3 spinal segments or roots (mostly ventral roots) were systematically stimulated, bilaterally, using handheld monopolar or bipolar electrodes while recording the MDP induced by each root stimulation, as previously described (19). We divided the spinal root evoked MDP data into L1–L6 and L7–S3 groupings, as in dogs, L7–S3 roots are the primary segments innervating the bladder (13, 17, 20), and the obturator nerve primarily originates from L3–L6 spinal cord segments in a variable range (21–24). The resulting maximum detrusor pressure (MDP) in cmH_2_O is reported.

### Ex Vivo Bladder Smooth Muscle Strip Contractility Studies

The ex vivo bladder smooth muscle strip contractility studies were described in Part 1 of this series (18).

### Tissue Collection

After the terminal surgeries, full thickness bladder specimens (∼3 cm^2^ in size) were also collected for histological studies. These specimens were collected from middle bladder regions located ventromedial to the ureteral orifices, between the dome and ∼2 cm above the bladder neck. Transferred obturator nerves were also collected at this time. Samples were fixed in 4% paraformaldehyde for 24 h and equilibrated in 10% sucrose in phosphate buffer for 24–48 h, followed by 30% sucrose in phosphate buffer for 24–48 h, before being embedded and frozen on dry ice in OCT Compound (Scigen, Gardena, CA), and stored at −80°C for future cryosectioning. The frozen tissue blocks of full bladder thickness specimens and nerves were cut into 14-µm sections and placed onto charged glass slides (Fisher, SuperFrost Plus, Pittsburgh, PA). Sections on slides were stored at −80°C until used for histological and immunohistochemical assays.

### Bladder Tissue Histology and Immunohistochemical Quantification

Bladder histological studies were performed on 14 Sham/Unop C, four Decentralized, and eight ObNT Reinn animals. Histological assays of the bladder wall sections were performed after hematoxylin and eosin (H&E) or Masson’s Trichrome staining, or after pgp9.5 or tyrosine hydroxylase immunohistochemistry. To visualize axons, immunohistochemistry was performed on subsets of sections (on slides) after being permeabilized with 0.5% pepsin for 15 min, blocked using 10% goat serum for 30 min, and incubated with a monoclonal antibody against pgp9.5 (a pan neuronal marker, no. ab8189, Abcam, Cambridge, MA), diluted 1:100 with phosphate buffered saline (PBS) overnight at room temperature. Slides were washed on a shaker with PBS and incubated with a goat anti-mouse antibody tagged with Cy3 (red; no. 111–165-166, Jackson ImmunoResearch, West Grove, PA) diluted 1:100 with PBS for 2 h at room temperature. Tyrosine hydroxylase immunohistochemistry was performed as previously described (19), after 0.3% Triton-X 100 permeabilization with an anti-rabbit tyrosine hydroxylase (no. JC1693653, Millipore, Burlington, MA), followed by a goat anti-rabbit secondary antibody tagged with AF488 (no. 111–645-144, Jackson ImmunoResearch). Specificity of these antibodies has been previously reported (19, 25).

Sections of tissues were imaged, and parameters quantified using either a Nikon Eclipse E600 microscope equipped with a digital camera (Nikon DS-Ri2, Nikon) and imaging software (NIS-Elements, Nikon Instruments, Melville, NY), or with a Nikon Eclipse E800 microscope equipped with an EXi Q-Imaging camera (QImaging, Surrey, BC, Canada) and imaging software (Bioquant Life Science, Bioquant Image Analysis Corp., Nashville, TN). A minimum of three bladder sections was examined per parameter, stain, and animal. Most assays were performed by 2–3 individuals, after a series of consensus training sessions and interim statistical analysis, with modification or clarification of counting rules until interobserver concordance was reached. Individuals performing the histological quantifications were blinded to the animals’ group assignment.

Adjacent H&E-stained bladder sections were used to assay the thicknesses of each bladder wall layer, area occupied by each layer per section, urothelium integrity, presence of inflammation, and intramural ganglia. In detail, thicknesses of the urothelium, submucosa, detrusor muscle layer, and bladder wall full thickness were assayed in multiple sites per layer: 10 different sites per urothelium, 4–9 sites per detrusor muscle layer, and 3–6 sites per submucosa layer and bladder wall full thickness. Urothelium integrity was assayed in at least two sections per animal, and scored as 0 = fully attached, normal height and no indices of inflammation or damage; 1 = thinning but fully attached; 2 = thinning and with partial detachment; and 3 = thinning and detached or mainly detached. Presence of inflammation was scored in the submucosa and detrusor muscle layer by examining a minimum of three H&E-stained sections per animal, with the score of 0 = no inflammatory cells, 1 = low numbers of lymphocytes but no neutrophils, 2 = moderate numbers of inflammatory cells (mainly lymphocytes), and 3 = large numbers of inflammatory cells, both lymphocytes and neutrophils, throughout or in dense clumps. The number of neuronal cell bodies per intramural ganglion profile (i.e., each sectioned profile) was quantified and normalized to the area of that ganglion. The number of intramural ganglia was normalized to the area of the full bladder wall in each section in which they were identified, and the number of neurons per ganglion area was also normalized to the area of the full bladder wall. An “exhaustive” method was used for the intramural ganglia quantification (3 people examined 3–6 H&E-stained sections per animal, in their entirety for the presence of any ganglia and imaged any observed ganglia for later neuronal cell body counts and quantification of the ganglion’s area).

Masson’s Trichrome-stained bladder sections were used to assay the percent area of the submucosa and detrusor muscle layers occupied by collagen (blue) and the percent area of the detrusor muscle layer occupied by muscle (reddish in color). For this, a thresholded color pixel count was used, with an irregular region of interest, and previously described imaging methods (26). An average of 10 different muscle sites were assayed per animal in 3–4 sections per animal. The number of pixels with collagen or muscle was normalized to the total number of pixels in the region of interest. Masson’s Trichrome-stained sections were also used to verify detrusor muscle layer and bladder wall full thicknesses.

After pgp9.5 and tyrosine hydroxylase immunostaining, the density of immunopositive axons was quantified in the detrusor muscle layer at ×300 magnification. Axon density was assayed using a previously described stereological grid count method (27). At least four randomly chosen fields were quantified per detrusor muscle layer, per animal, with data normalized to the area of the region of interest (0.28 mm^2^).

### Obturator Nerves

Cryosectioned obturator nerves were stained with H&E or were coverslipped with 80% glycerol in phosphate buffer only. Sections were examined microscopically (×10–×200 magnification) for integrity of the transferred nerves and presence of fluorogold labeled axons proximal to the anastomosis site, as previously described (14).

### Statistical Analyses

Statistical analyses were performed using Prism 8 (GraphPad Software, La Jolla, CA). All data are presented as means and 95% confidence intervals (CI), and the level of significance was set at *P* < 0.05 for all analyses (adjusted *P* values are reported). Note, this study did not test a prespecified statistical null hypothesis, which makes it exploratory by default. Thus, it follows that any calculated *P* values can only be interpreted as descriptive, not as hypothesis testing. The majority of the statistical intergroup comparisons were defined before the results were observed.

Both Shapiro–Wilk and Kolmogorov–Smirnov tests of normality were performed first and residuals inspected. For data that were not normally distributed, or for data that consisted of histological scores, a Kruskal–Wallis ANOVA was used to compare the three groups. Kruskal–Wallis ANOVAs were followed by Dunn’s multiple comparison post hoc tests. Otherwise, Brown–Forsyth one-way ANOVAs were used to compare findings across three groups, followed by Dunnett’s multiple comparisons post hoc tests. A repeated-measures mixed-effect model (REML, REstricted Maximum Likelihood model) was used for the spinal root stimulated maximum detrusor pressure data using two factors [surgical group and segment (L1–L6 vs. L7–S3], followed by a Tukey’s multiple comparisons post hoc test. Pearson’s or Spearman rank correlation tests were used for correlations, as appropriate for the data, with *r*_p_ and *r*_s_ findings reported in figure panels. Values between 0.4 and 0.59 (−0.4 and −0.59) were considered as a moderately positive (or negative) relationships, values between 0.6 and 0.79 (−0.6 and −0.79) as a strongly positive (or negative) relationships, and 0.8–1.0 as very strong correlations (28, 29). Only moderate to very strong correlations were considered and reported, although these are rather arbitrary limits and the context of the results should be considered.

## RESULTS

### Observations following Surgery

Incidence of culture confirmed bacteriuria and antibiotic treatment were described in Part 1 of this series (18).

### Urothelium Integrity Worsens as the Submucosa Inflammation Score Increases

The urothelium thickness, when present, did not differ across groups (ANOVA, *P* = 0.15, Fig. 1*A*). However, there were changes in the urothelium integrity and submucosa inflammation scores (ANOVAs, *P* = 0.008 and *P* = 0.001, respectively, Fig. 1, *B* and *C*). Post hoc assays showed that the urothelium was thinning and partially detached (scored as 1.5 to 2) in all of the Decentralized animals and most of the ObNT Reinn animals compared with Sham/Unop C animals (Fig. 1*B*). One ObNT Reinn animal showed complete urothelium detachment (scored as 3), whereas another showed a fully intact urothelium (scored as 0, Fig. 1*B*). The Decentralized and ObNT Reinn animals showed higher inflammation scores in the submucosal layer compared with Sham/Unop C (Fig. 1*C*). Worse (higher) urothelium integrity scores correlated strongly with higher scores of submucosa inflammation (Fig. 1*D*), supporting a role of inflammation in the urothelium pathology, or vice versa (i.e., a role of urothelium pathology in submucosal inflammation). Representative images are shown in Fig. 1, *E–I*. Images from two different Decentralized animals (Fig. 1, *F* and *G*) and three different ObNT Reinn animals (Fig. 1, *H* and *I* and *inset* in Fig. 1*H*) are shown to provide a sense of the range in urothelium integrity and inflammation observed. Lymphocyte infiltration was common, although one Decentralized animal also had more neutrophils. A few animals showed large clumps of lymphocytes in the submucosa (e.g., Fig. 1*I*). More blood vessels were present in the submucosa of Decentralized and ObNT Reinn animals (Fig. 1, *F*) than in Sham/Unop C animals (Fig. 1*E*).

### Long-Term Decentralization, with or without Reinnervation, Results in Thickening and Inflammation in Deeper Layers

The thickness (height) of the submucosal layer did not differ across groups (Brown–Forsyth ANOVA, *P* = 0.22, Fig. 2*A*). However, the detrusor muscle layer and bladder wall full thickness differed between groups (ANOVAs, *P* = 0.01 and *P* = 0.02, respectively, Fig. 2, *B* and *C*), as did detrusor muscle inflammation scores (ANOVA, *P* = 0.001, Fig. 2*D*). Post hoc analyses showed that the detrusor muscle layer was thicker in Decentralized and ObNT Reinn animals compared with Sham/Unop C (Fig. 2*B*). The full bladder wall was thicker in both Decentralized and ObNT Reinn animals compared with Sham/Unop C (Fig. 2*C*). The detrusor muscle layer of Decentralized and ObNT Reinn animals had higher inflammation scores compared with Sham/Unop C (Fig. 2*D*). The increase in connective tissues surrounding smooth muscle layers in Decentralized and ObNT Reinn versus Sham/Unop C bladders can be visualized in representative low power images (Fig. 2, *E*–*G*). Higher power images show examples of more neutrophils and lymphocytes in the connective tissues surrounding detrusor muscle bundles, nerves, and invading intramural ganglia of Decentralized and ObNT Reinn animals, than were observed in Sham/Unop animals (Fig. 2, *I* and *J* and *insets*). Many of the neuronal cell bodies in the intramural ganglia were pyknotic in Decentralized and ObNT Reinn animals (examples shown in Fig. 2*J*, *inset*).

### Long-Term Decentralization, with or without Reinnervation, Results in Increased Collagen, Although Percent Muscle is Maintained

**Figure 3.**
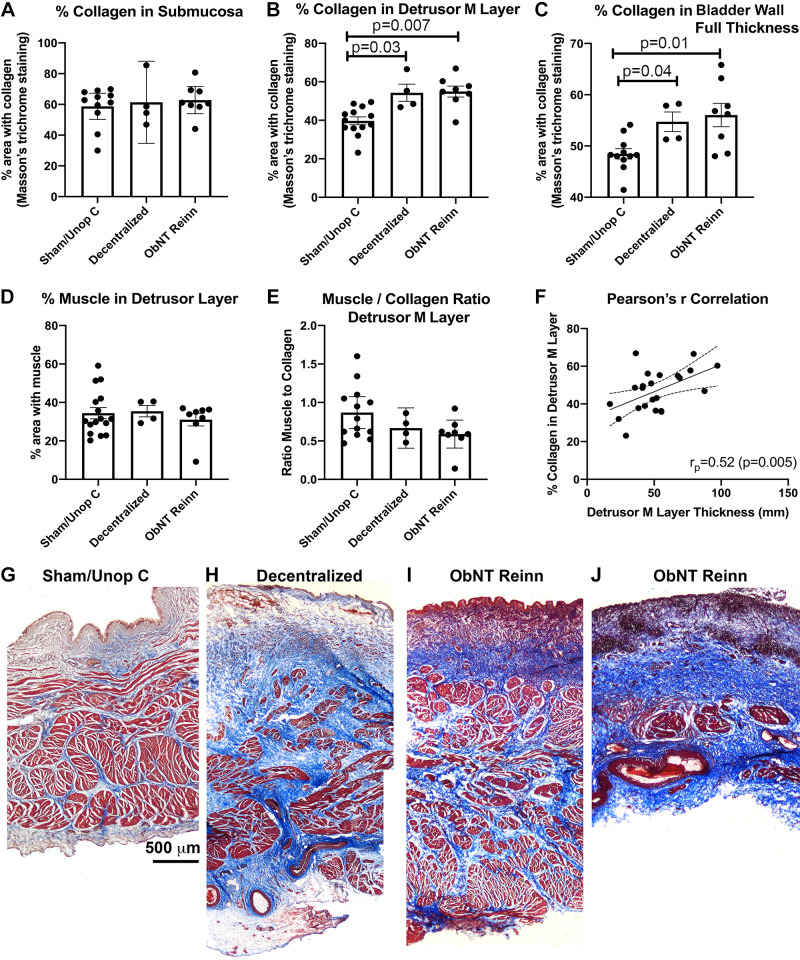
Percent collagen and percent muscle quantified after Masson’s Trichrome staining. *A–C*: percent collagen (stained blue in panels *G*–*J*) in the submucosa and detrusor muscle (M) layers, and bladder wall full thickness. *D*: percent muscle (stained red in panels *G*–*J*) in the detrusor muscle layer. *E*: ratio of muscle to collagen staining in the detrusor muscle layer. Means and 95% CI are shown in panels *A–E*. *F*: Pearson’s *r* correlation (*r*_p_) between percent collagen detrusor muscle layer and its thickness. The linear regression line and 95% confidence intervals are shown. *G–J*: representative images of each group, with two images provided for the ObNT Reinn group since one animal had very low percent muscle. Kruskal–Wallis ANOVAs were used for analyses of collagen in the submucosal layer and the muscle to collagen ratio and Brown–Forsyth ANOVAs were used for analyses of collagen in detrusor muscle layer and full bladder wall. Scale bar in *G* is applicable to *H–J*. CI, confidence interval.

Masson’s Trichrome staining showed no change in collagen deposition in the submucosal layer across groups (ANOVA, *P* = 0.79, Fig. 3*A*) yet did show more collagen in detrusor muscle layer and full bladder wall (ANOVAs, *P* = 0.004 and *P* = 0.01, respectively, Fig. 3, *B* and *C*). Post hoc analyses showed increases in the percent area with collagen staining in the detrusor muscle layer of Decentralized and ObNT Reinn animals compared with Sham/Unop C (Fig. 3*B*), and increases in percent collagen in the bladder wall full thickness of Decentralized and ObNT Reinn animals compared with Sham/Unop C (Fig. 3*C*). The percent area with muscle in the detrusor muscle layer did not differ across groups (ANOVA, *P* = 0.76, Fig. 3*D*). The muscle to collagen ratio differed slightly between groups (ANOVA, *P* = 0.04, Fig. 3*E*), although there were no statistically important post hoc test findings, most likely because one ObNT Reinn animal showed an extreme loss of muscle. Detrusor muscle layer thickness correlated moderately and positively with the percent collagen in that layer (Fig. 3*F*). Representative images are shown in Fig. 3, *G*–*I*, and Fig. *3J* shows the extreme loss of muscle in the one ObNT Reinn animal mentioned earlier.

### Long-Term Decentralization, with or without Reinnervation, Results in a Loss of Intramural Ganglia

**Figure 4.**
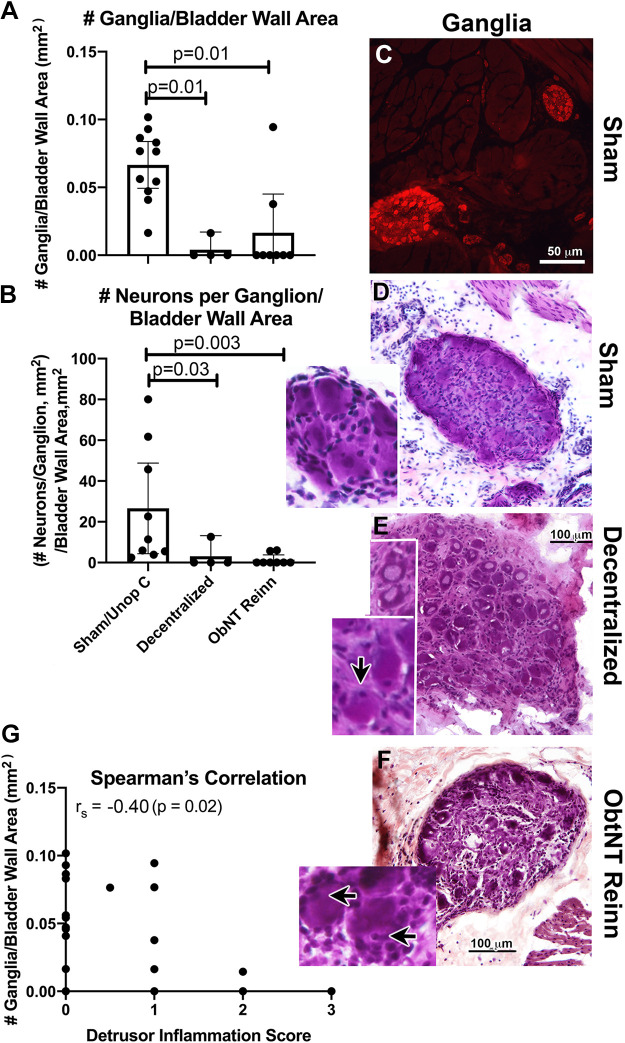
Assessment of intramural ganglia in the bladder wall. *A* and *B*: number of ganglia and number of neurons/intramural ganglia area, each normalized to the full bladder wall area. Means and 95% CI are shown. *C*: example of intramural ganglia in a sham animal, after pgp9.5 immunostaining (red color). *D–F*: examples of intramural ganglia from each group (when present) after H&E staining. *Insets* show higher power images of ganglia, with centrally located nuclei in a sham animal (*D*), yet eccentrically located nuclei in Decentralized and ObNT Reinn groups (indicated with arrows in the *insets* of *E* and *F*). *G*: Spearman’s rank correlation (r_s_) between number of ganglia per full bladder wall area and detrusor muscle layer inflammation score. Kruskal–Wallis ANOVAs were used for analyses of numbers of intramural ganglia and neurons in them. Scale bars in *E* and *F* are applicable to *D*. CI, confidence interval.

The number of intramural ganglia, after normalization to the full bladder wall area, differed across groups, as did the neurons/ganglia, after normalization to the full bladder wall area, (ANOVAs, *P* = 0.02 and *P* = 0.002, respectively). Post hoc analyses showed that the number of intramural ganglia/full bladder wall area was considerably lower in Decentralized animals and ObNT Reinn animals compared with Sham/Unop C animals (Fig. 4*A*), as was the number of neurons per ganglia after normalization to the full bladder wall area (Fig. 4*B*). Representative images of intramural ganglia (when found) are shown in Fig. 4, *C*–*F*. Intramural ganglia in sham animals were healthy looking and contained many neurons (Fig. 4, *C* and *D*), with central nucleated neurons (Fig. 4*D*, *inset*). In contrast, intramural ganglia in Decentralized and ObNT Reinn animals, when found, were chromatolytic (pale and swollen nuclei) or pyknotic with eccentrically located nuclei (Fig. 4, *E* and *F* and *insets*). The number of ganglia per bladder wall showed a moderate and negative correlation with detrusor muscle layer inflammation scores (Fig. 4*G*).

### Submucosal Inflammation May Also Be Contributing to Detrusor Histological Changes

**Figure 5.**
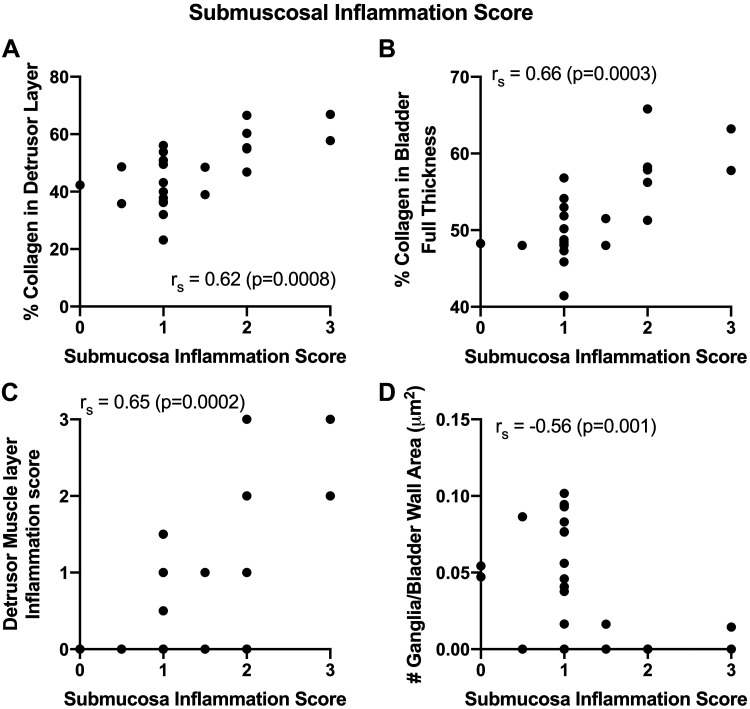
Correlations between submucosal inflammation scores and histological findings. Spearman’s rank correlations (*r*_s_) were performed to examine associations between submucosal inflammation scores and the % collagen in the detrusor muscle layer thickness (*A*), % collagen in the bladder wall full thickness (*B*), detrusor muscle layer inflammation score (*C*), and number of ganglia per bladder wall area (*D*).

The earlier presented submucosa inflammation scores (Fig. 1*C*) correlated strongly and positively with percent collagen in both the detrusor muscle layer and in the full bladder wall with the (Fig. 5, *A* and *B*), and with the detrusor muscle layer inflammation score (Fig. 5*C*). This score also showed a moderate and negative correlation with the number of ganglia per bladder wall (Fig. 5*D*). Combined, these findings support a contribution of submucosal inflammation to detrusor muscle layer and bladder wall pathology.

### Reinnervation Leads to a Higher Density of pgp9.5 Immunopositive Axons in ObNT Reinn Animals

**Figure 6.**
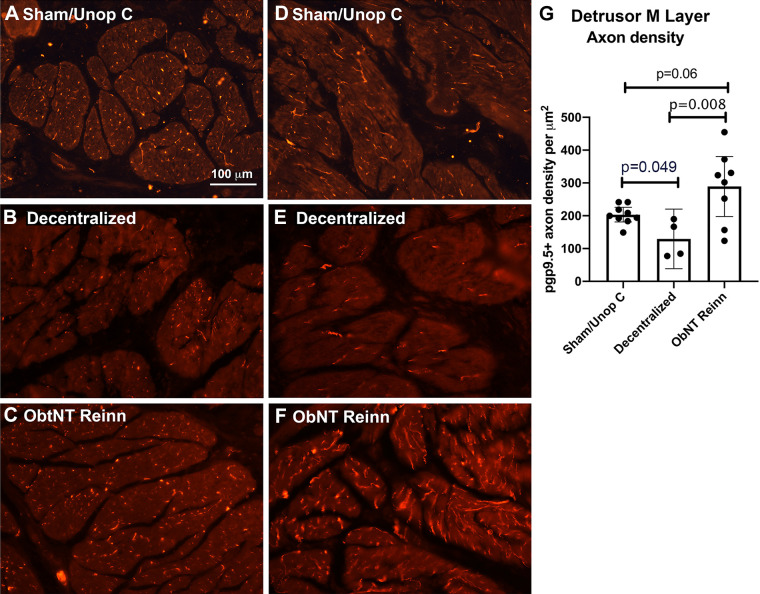
Pgp9.5 immunostained axons in the detrusor muscle layer. *A–F*: representative images of pgp9.5 immunostained axons (red) in cross-sectional bundles of the detrusor muscle (*A–C*) and in longitudinal bundles of the detrusor muscle (*D–F*). Scale bar in *A* is applicable to *B–F*. *G*: quantification of pgp9.5 immunostained axon density. A Brown–Forsyth ANOVA was used. Means and 95% CI are shown. CI, confidence interval.

In the pilot study (14) and Part 1 of this series (18), the bladders of ObNT Reinn animals showed changes in in vivo and ex vivo contractility. Thus, we next performed immunostaining for pgp9.5 (a pan neuronal marker) and assessed the density of axons in the detrusor muscle layer (Fig. 6). We were able to detect axons as small as 0.09 microns in width [the mean size of all axons counted was 1.41 (95% CI: 1.25–1.53), ranging from 0.09 to 5.7 microns in width]. Although there were no differences between groups in mean axon width, we observed differences across the groups in pgp9.5 immunopositive (+) axon density in the detrusor muscle layer (ANOVA, *P* = 0.01, Fig. 6, *A*–*F*). Post hoc analysis showed a decline in pgp9.5+ axon density in both cross-sectional and longitudinal smooth muscle bundles in Decentralized animals compared with Sham/Unop C animals (Fig. 5, *B* vs. *A* and Fig. 5, *E* vs. *D*; quantification shown in Fig. 6*G*). In contrast, pgp9.5+ axon density was increased in ObNT Reinn animals compared with Decentralized animals (Fig. 6, *C, F* vs. *B*, *E*; quantification shown in Fig. 6*G*). The pgp9.5+ axon density in ObNT Reinn animals was comparable to that seen in Sham/Unop C animals (*P* = 0.06, Fig. 6*G*).

### Extensive Decentralization and Reinnervation Surgery Reduced Tyrosine Hydroxylase Immunopositive Axons

**Figure 7.**
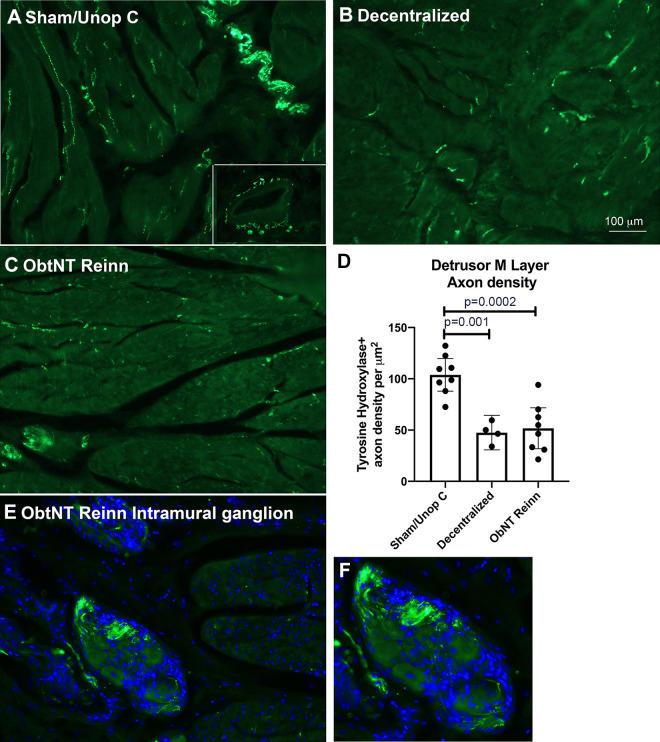
Tyrosine hydroxylase immunostained axons in the detrusor muscle layer. *A–C*: representative tyrosine hydroxylase immunostained axons (green) in cross-sectional bundles of the detrusor muscle from each group. The *inset* in *A* shows tyrosine hydroxylase staining around an artery. *D*: quantification of tyrosine hydroxylase immunostained axon density. A Brown–Forsyth ANOVA was used. Means and 95% CI are shown. *E*: an intramural ganglion in an ObNT Reinn animal (enlarged in *F*). The neuronal cell bodies did not express tyrosine hydroxylase. Scale bar in *B* is applicable to *A*, *C*, and *E*. CI, confidence interval.

As there are reports of sympathetic axonal sprouting after sacral decentralization of the bladder (30–32), and even spontaneous regrowth of the hypogastric nerve after long-term transection (33), we examined the density of tyrosine hydroxylase immunostained axons in the detrusor muscle layer (Fig. 7). Microscopic examination (Fig. 7, *A*–*C*) and quantification showed differences across groups in tyrosine hydroxylase+ axon density in the detrusor muscle layer (ANOVA, *P* < 0.0001). Post hoc analysis showed declines in tyrosine hydroxylase + axons in Decentralized and ObNT Reinn animal bladders compared with Sham/Unop C animals (Fig. 7, *A*–*D*). An intramural ganglion in the bladder wall from an ObNT Reinn animal shows no tyrosine hydroxylase+ neurons (Fig. 7*E*). These results indicate that sympathetic sprouting or regrowth is not contributing to the overall increase in pgp9.5+ axon density.

### Ex Vivo Detrusor Smooth Muscle Strip Responsiveness Appears To Be Related to Inflammation and Innervation Changes

**Figure 8.**
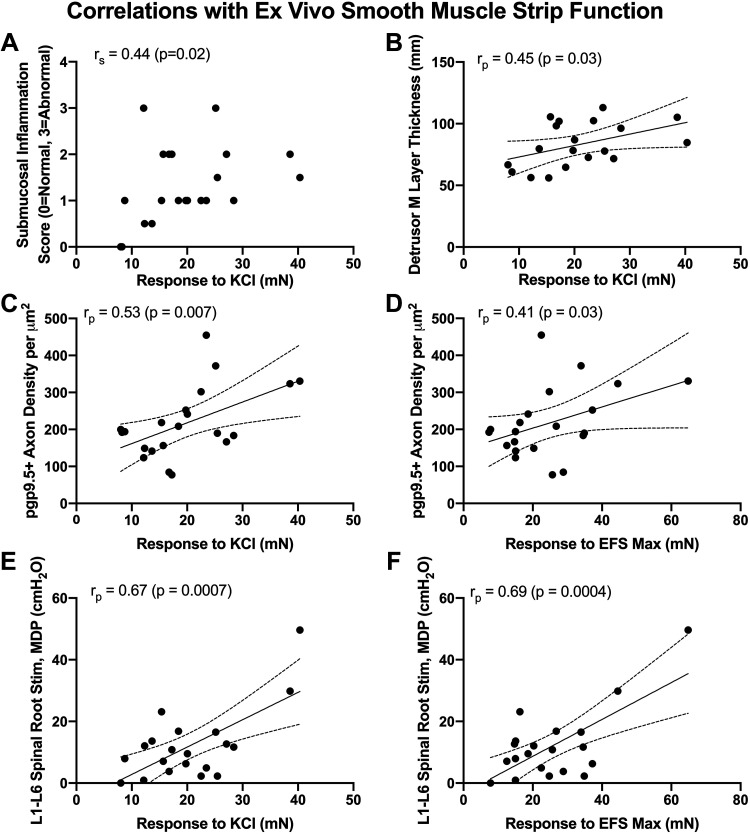
Moderate to strong correlations between ex vivo smooth muscle strip contraction findings and histological or in vivo functional findings. Pearson’s *r* correlations (*r*_p_) were performed to examine these associations. *A*: the contractile response to muscle strips to KCl (in mN) vs. submucosal inflammation scores. *B*: the contractile response of muscle strips to KCl (mN) vs. detrusor muscle (M) layer thickness. *C*: the contractile response of muscle strips to KCl versus pgp9.5 axonal density in the bladder wall. *D*: the contractile response to maximum electric field stimulation (EFS Max in mN) versus pgp9.5 axonal density in the bladder wall. *E*: the contractile response of muscle strips to KCl (mN) versus *in vivo* responses of the bladder to L1-L6 root stimulation. *F*: the contractile response of muscle strips to EFS Max versus *in vivo* responses of the bladder to L1-L6 root stimulation. Linear regression lines and 95% CI are shown. CI, confidence interval.

Ex vivo muscle strip responsiveness data presented in Part 1 of this work (18) was correlated with both the histological and in vivo electrophysiological findings. Responsiveness of detrusor smooth muscle strips to KCl treatment showed moderate and positive correlations with submucosal inflammation scores, detrusor muscle layer thickness, and pgp9.5+ axon density in the detrusor muscle layer (Fig. 8, *A*–*C*). Yet, the responsiveness of detrusor smooth muscle strips to KCl treatment did not correlate with percent collagen deposition (fibrosis) in the detrusor muscle layer (*r*_p_ = 0.09, *P* = 0.34), suggesting that the correlation with detrusor muscle layer thickness was due to the percent muscle instead. The responsiveness of detrusor smooth muscle strips to maximum electric field stimulation also showed a moderate and positive correlation with pgp9.5+ axon density in the detrusor muscle layer (Fig. 8*D*). Strong positive correlations were also observed between the responsiveness of detrusor smooth muscle strips to KCl treatment and maximum electric field stimulation and in vivo MDPs evoked during L1–L6 root stimulations (Fig. 8, *E* and *F*).

### Nerve-Evoked In Vivo Bladder Contractility Is Reduced after Long-Term Decentralization, Likely Due to Bladder Fibrosis and Chronic Inflammation

**Figure 9.**
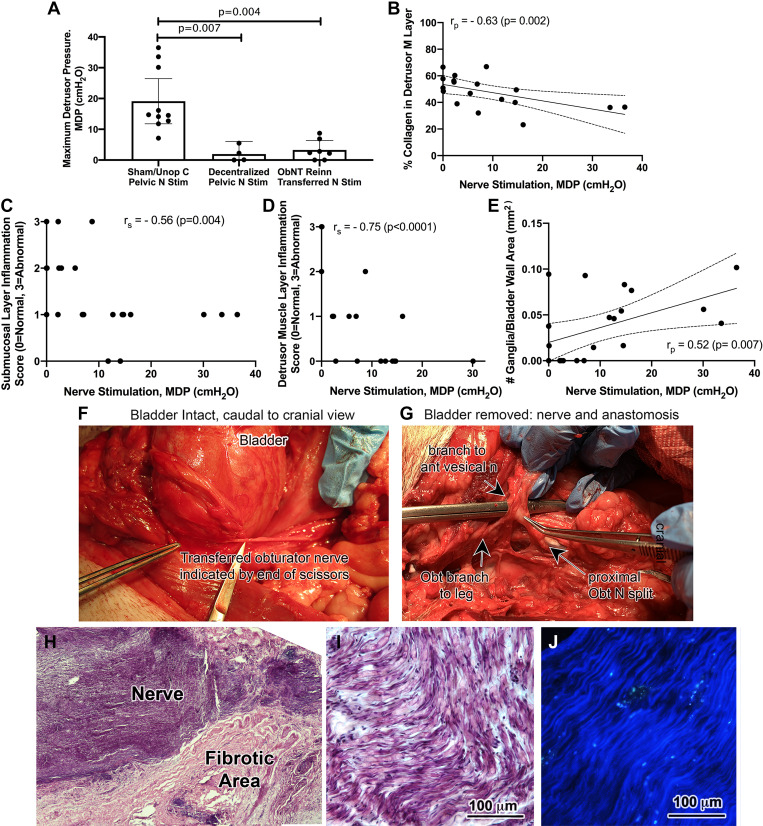
Detrusor responsiveness to pelvic nerve stimulation and its correlation to histological findings. *A*: maximum detrusor pressure (MDP, cmH_2_O) generated after pelvic nerve stimulation in Sham/Unop C, or after obturator nerve stimulation in Decentralized and ObNT Reinn. A Kruskal–Wallis ANOVA was used. Means and 95% CI are shown. *B*: Pearson’s rank correlation between nerve-evoked MDP and the percent collagen in the detrusor muscle layer. *C* and *D*: Spearman’s rank correlations (*r*_s_) between nerve-evoked MDP and the submucosal and detrusor muscle layer inflammation scores. *E*: Pearson’s rank correlation between nerve-evoked MDP and the number of intramural ganglia per full bladder wall area. The linear regression line and 95% confidence intervals are shown when Pearson’s correlations were used. *F–J*: images showing the transferred obturator nerve. *F*: a lateral-to-medial view of a transferred obturator nerve distal to the still intact bladder during the terminal surgery. *G*: a midline-to-lateral view of a transferred obturator nerve after removal of the bladder. The intact obturator nerve is shown on the right side of the panel, the split off branch coapted for anastomosis to the anterior vesicle branch of the pelvic nerve is shown in the middle, and the remaining branch to the abductor muscles of the thigh is indicated on the left lower side of this panel. *H*: low power image of an obturator nerve near the coaptation site (×10 magnification) showing extensive fibrosis. H&E staining. *I*: higher power image of the obturator nerve at the coaptation site showing myelinated axons in a longitudinal cut section. H&E staining. *J*: the presence of fluorogold retrograde labeling in axons within the obturator nerve at a site proximal to anastomosis site, after injection of the dye into bladder. CI, confidence interval.

MDP after pelvic nerve stimulation, or stimulation of the transferred obturator nerve proximal to the site of anastomosis, was recorded during the terminal surgery and showed differences between the surgical groups (ANOVA, *P* < 0.0001, Fig. 9*A*). Post hoc analysis showed that the Decentralized animals had a decrease in pelvic nerve-induced MDP compared with pelvic nerve stimulation in Sham/Unop C animals (Fig. 9*A*), most likely due to the L7–S3 root transections. Two of the four Decentralized animals showed no bladder contractions after pelvic nerve stimulation (Fig. 9*A*). Although the ObNT Reinn animals showed a better outcome after stimulation of the transferred obturator nerve at a site immediately proximal to surgical anastomosis (5 of 7 animals showed bladder contractions after this stimulation), their nerve-evoked MDPs were still lower than in Sham/Unop C animals (Fig. 9*A*). Nerve-induced MDP contractions correlated strongly and negatively with percent collagen in the detrusor muscle layer (Fig. 9*B*), negatively with the submucosal and detrusor muscle layer inflammation scores (Fig. 9, *C* and *D*; strongly with the latter), and moderately and positively with the number of ganglia in the bladder wall (Fig. 9*E*). We speculate that repair-induced fibrosis around the transferred nerve and anastomosis site contributed to the low nerve-induced MDP contractions in reinnervated animals, as this fibrotic tissue made it difficult to find and stimulate the nerves. This hypothesis is supported by the difficulty in finding the transferred nerve site in one ObNT Reinn animal due to extensive fibrotic tissue (no stimulation data could be collected). Figure 9, *F–J* shows examples of transferred obturator nerves at the time of euthanasia. In several, the transferred obturator nerve could be identified at the base of the bladder proximal to its surgical coaptation site to the anterior vesicle branch of the pelvic nerve (Fig. 9*F*). Figure 9*G* shows the split in the obturator nerve site after bladder removal so that about one-quarter was coapted for the nerve transfer procedure and the remaining still innervated structures in the lower extremity. Pronounced extra neural fibrosis was often evident around this coaptation site (Fig. 9*H*). However, within the transferred nerves, intact and myelinated axons could be seen on microscopic examination (Fig. 9*I*), as could Fluorogold labeled axons [a transferred nerve is shown proximal to the coaptation surgical site in Fig. 9J and (14)].

### Spinal Root-Evoked In Vivo Bladder Contractility Is Reduced in Caudal Lumbar/Sacral Segments after Decentralization, yet Higher in More Cranial Lumbar Segments in ObNT Reinn Animals

**Figure 10.**
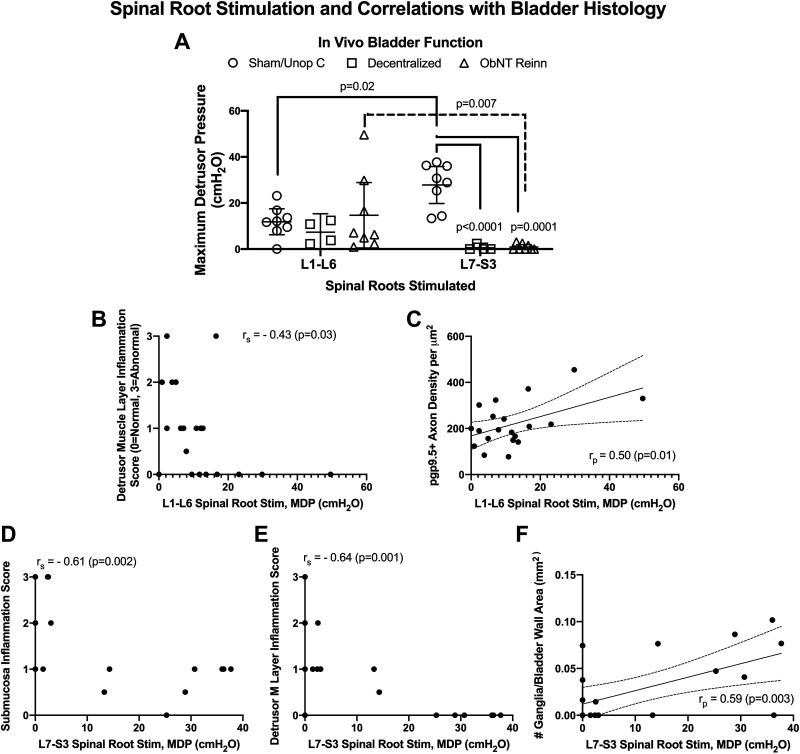
Detrusor responsiveness to spinal root stimulation and its correlation with histological findings. *A*: maximum detrusor pressure (MDP, cmH_2_O) generated after stimulation of L1–L6 and L7–S3 spinal roots. A repeated-measures mixed-effect model analysis was used. Means and 95% CI are shown. *B*: Spearman’s rank (*r*_s_) correlations between L1–L6 spinal root-evoked MDP and the detrusor muscle layer inflammation score. *C*: Pearson’s *r* correlation (*r*_p_) between L1–L6 spinal root-evoked MDP and pgp9.5 immunostained axon density. *D* and *E*: Spearman’s rank (*r*_s_) correlations between L7–S3 spinal root-evoked MDP and submucosal and detrusor muscle layer inflammation scores. *F*: Pearson’s *r* (*r*_p_) correlations between L7–S3 spinal root-evoked MDP and the number of intramural ganglia per full bladder wall area. Linear regression lines and 95% confidence intervals are shown when Pearson’s correlations were used. CI, confidence interval.

At terminal surgeries, MDP was recorded after stimulation of L1 through S3 spinal roots, and the results divided into L1–L6 and L7–S3 segmental outcomes (Fig. 10*A*). A repeated-measures mixed-effect model analysis showed a surgical group × segmental group interaction (*P* = 0.007) and a surgical group effect (*P* = 0.02). Post hoc analyses showed that after stimulation of L7–S3 spinal roots, MDP was considerably higher in Sham/Unop C animals compared with L7-S3 stimulation results in Decentralized and ObNT Reinn animals, confirming decentralization in the latter two groups (Fig. 10*A*). Importantly, after stimulation of L1–L6 roots, MDPs were higher in ObNT Reinn animals compared with their L7–S3 MDPs, and lower in Sham/Unop C animals compared with their L7–S3 stimulation results (Fig. 10*A*). MDP evoked after L1–L6 root stimulation correlated moderately and negatively with inflammation in the detrusor muscle layer (Fig. 10*B*) and moderately and positively with pgp9.5+ axon density (Fig. 10*C*). Last, MDPs evoked after L7–S3 root stimulation correlated strongly and positively with the submucosal and detrusor muscle layer inflammation scores (Fig. 10, *D* and *E*), and moderately and positively with the number of intramural ganglia in the bladder wall (Fig. 10*F*).

## DISCUSSION

We show here for the first time that 11–21 mo Decentralized and ObNT Reinn (9–13 mo decentralized and then 8–12 mo recovery after nerve transfer) animals show partial to full urothelium detachment, inflammation in submucosa and detrusor muscle layers, thicker bladder walls due to enhanced collagen deposition primarily in the detrusor muscle layer, and reduced intramural ganglia and intramural neurons compared with Sham/Unop C animals. Yet, we also observed greater pgp9.5+ axon density in ObNT Reinn animals than in Decentralized animals (and equivalent to Sham/Unop C animals) and preservation of the percent smooth muscle across groups. The latter finding supporting our prior report that bladder smooth muscle does not atrophy after long-term decentralization. Functionally, we also found that ex vivo smooth muscle strip contractility in response to KCl treatment correlated positively with submucosa inflammation, detrusor muscle layer thickness, and pgp9.5 axon density (the latter also correlated with muscle strip contractility in response to maximum electric field stimulation). Also, muscle strip contractility in response to KCl and maximum electric field stimulation correlated positivity with MDP after L1-L6 spinal root stimulation. Together, this suggests that submucosal inflammation may be enhancing muscle strip contractile responsiveness to KCl, and that either the maintained muscle or pgp9.5 axon density, or both, contributed to the smooth muscle contractile responses. In vivo nerve-evoked MDP was decreased in Decentralized and ObNT Reinn animals, respectively, compared with nerve stimulation in Sham/Unop C animals, findings that correlated negatively with collagen deposition (i.e., fibrosis) and inflammation in the submucosal and detrusor muscle layers, and positively with intramural ganglia numbers. Spinal root-evoked MDP was expectedly decreased in Decentralized and ObNT Reinn animals after stimulation of the long-transected L7–S3 roots compared with Sham/Unop C animals, confirming their decentralization. However, spinal root-evoked MDP was considerably higher in ObNT Reinn animals after stimulation of their L1–L6 roots compared with their L7–S3 roots. This correlated positively with pgp9.5+ axon density in the detrusor layer and negatively with inflammation in the submucosal and detrusor muscle layers. Combined, these findings suggest that innervation (either as input from the spinal cord through intact or transferred nerves, pgp9.5 axonal regrowth, or maintained intramural ganglia) is key to bladder function, and that fibrosis and persistent inflammatory responses can be detrimental.

The decentralization procedure required for this study necessarily included bilateral transection of all roots caudal to L7 and the dorsal roots of L7, as well as the hypogastric nerves, in order to eliminate micturition behaviors that persisted after sacral root decentralization only (14). Many of the sympathetic/adrenergic efferents and visceral afferents that are involved in micturition behaviors are carried by the hypogastric nerves, which originate from L1 to L5 spinal cord segments in dogs (22, 34). Sympathetic axons can sprout in the bladder after sacral nerve decentralization only (30–32) and even regrow after hypogastric nerve transection and removal (33). We did not observe such sprouting or regrowth of sympathetic axons after the extensive decentralization or reinnervation procedures. We believe that this extent of decentralization reproduces the maximum dysfunction experienced by a patient with lower motoneuron neurogenic bladder. It also allowed for a clear appreciation of the effects of prolonged extensive decentralization on bladder wall integrity (and issues thereof) and aided the interpretation of the obturator nerve transfer results.

We observed that stimulation of L1–L6 spinal roots has some effects on bladder pressure in Decentralized animals, more in Sham/Unop animals (although less than after stimulation of L7–S3 roots in Sham/Unop animals, Fig. 10*A*) and even more in animals in which a lumbar originating nerve, the Obturator, was transferred to the bladder (Fig. 10*A*). There are several motor projections to the bladder from lumbar ventral horns that do not involve hypogastric nerves (which were transected in Decentralized and ObtNT Reinn animals). A number of sympathetic preganglionic neurons in lumbar ventral horns project to postganglionic neurons located in sacral sympathetic trunk ganglia (35), these then send motor input to the bladder via sacral splanchnics (17, 20, 36). We have shown that the number of labeled neurons projecting to the bladder from sacral sympathetic trunk ganglia may be lower after sacral root transections, yet still present (20). The bladder also receives both indirect and direct preganglionic motor projections from upper and mid lumbar ventral horns via lumbar splanchnics (17, 20, 37). Direct lumbar projections to the bladder have been identified using retrograde dye labeling methods (17, 20, 38). Functionally, we have reported that stimulation of L2 ventral roots evokes fairly strong contractions of the bladder after transection of L6 and L7 dorsal roots and all spinal roots caudal to L7, if the hypogastric nerve is left intact (explaining the bladder contractions after L1–L6 root stimulation in Sham/Unop animals) (19). Yet, in dogs, after transection of hypogastric nerves and all roots caudal to L5, low amplitude evoked contractions are still present during L2 spinal root stimulation (19), likely as a result of maintained direct or indirect inputs from the L2 ventral horn to the bladder. Perhaps a strength of our reinnervation strategy, in which we use lumbar originating nerves as donor nerves (6, 13, 14, 17), is that the lumbar region already contains at least a few motor neurons that innervate the bladder and thus some of the necessary motor components needed for bladder contraction.

Submucosal inflammation, observed mainly as lymphocyte infiltration and increased blood vessels in Decentralized and ObNT Rein animals, correlated strongly with increased partial to full detachment of the urothelium (Fig. 1*D*). This matches prior results from our laboratory of similar partial detachment of the urothelium and submucosal inflammatory responses in two of four animals that had been decentralized for 12 mo before tissue collection, after the same L7 dorsal and S1–3 dorsal and ventral root transection strategy (17). In this study, all Decentralized and ObNT Reinn animals had confirmed bacteriuria and were treated with antibiotics at the time of detection, repeatedly (∼244 days) throughout the experiment (which was up to 21 mo). Chronic inflammation can initiate apoptosis and disruption of the urothelial barrier, and can exacerbate inflammation in the submucosal and detrusor muscle layers (39–41). As inflammation in the upper bladder layers might also be in response to, rather than a cause of, bladder urothelial damage (42), a vicious cycle can result (41). Recently, Montalbetti and colleagues reported that increased urothelial permeability, even without desquamation, is sufficient to cause lymphocytic infiltration and edema in the urinary bladder as well as altered bladder function (reduced compliance and increased voiding frequency) as a result of afferent hyperactivity (43, 44). Inflammation can also induce hypersensitivity of afferents in the bladder (39, 45). Applicable to this study, there is evidence that decentralization alters urothelial integrity. For example, spinal cord injuries are associated with a number of urothelium changes, including defects in urothelium barrier function and signaling (46, 47). Thus, it is hard to say which comes first, defects in the urothelium or submucosal inflammation, or which reduces bladder compliance the most, since a reduction may develop secondarily to chronic inflammation or as a consequence of the decentralization-induced neuromorphological and neuropharmacological reorganization of the bladder (48). As there is evidence for each scenario in the literature, it is likely that all are contributing factors.

Detrusor muscle layer inflammation (increased lymphocytes and neutrophils) was observed in Decentralized and ObNT Reinn animals at 20–22 mo after decentralization. Detrusor muscle layer inflammation may be related to the observed submucosa inflammation since submucosa is an active tissue that regulates neuronal signaling, including possible signaling to the detrusor muscle layer (46, 49). Such persistent inflammation can contribute to decreased bladder compliance (40, 50), perhaps because proinflammatory mediators, such as proinflammatory cytokines, released by infiltrating immune cells are cytotoxic to muscles, nerves, and neuronal cell bodies, and contribute to Wallerian degeneration (51, 52). We see evidence of this in the lymphocyte-invaded nerve bundle and intramural ganglion (with pyknotic neuronal cell bodies) shown in Fig. 2, *I* and *J*, as well as degenerating neurons in ganglia shown in Figs. 3 and 4. We believe that inflammation-induced cytotoxicity and other signaling events [e.g., enhanced cellular apoptosis (53)] underlie the negative correlations seen between nerve- and L1–L6 root-evoked MDP contractions and detrusor muscle layer inflammation (Fig. 9*C* and Fig. 10*B*).

Chronic inflammation can also enhance collagen deposition in neuromuscular tissues in general (25, 54) and, specific to the bladder, induce collagen deposition between muscle cells in the detrusor muscle layer (40, 55). Collagen content also increases in the bladder wall as a consequence of neuronal decentralization (55). Such increases are known to reduce bladder tissue compliance and impair contractility (40, 55). Thus, fibrosis is a nonneurogenic contributor to bladder contractility dysfunction and persistent inflammatory responses can enhance that fibrosis. Fibrosis around the site of anastomosis of the obturator nerve to the anterior vesicle branch of the pelvic nerve (such as shown in Fig. 9*H*) may also be one reason for the lower MDP responses after direct stimulation of the transferred nerves (<10 cm H_2_0) than after L1–L6 spinal root stimulation (up to 50 cm H_2_0) as this buildup of fibrosis would block the electrical signal from the stimulator to the nerve (although also, as mentioned earlier, L1–L6 spinal root stimulation would also stimulate still intact lumbar originating sympathetic splanchnics and preganglionic sympathetics that would induce bladder contractions directly and indirectly). The combined negative correlations between nerve- and root-evoked MDP contractions and inflammation in the submucosal and detrusor muscle layers, and detrusor muscle layer collagen deposition (Fig. 9, *B*–*D*, and Fig. 10*, B, D,* and *E*) support a strong need to control either the chronic inflammation or fibrosis (or both), for improved reinnervation and function in patients with spinal root or cord damage.

We observed for the first time a loss of intramural ganglia and their neuronal numbers in these long-term and extensively decentralized animals compared with shorter studies or less decentralized bladders (16, 17, 56). Intramural ganglion neurons are postganglionic neurons located in the bladder wall of dogs, cats, guinea pigs, and humans (not rats or mice) between detrusor smooth muscle bundles (17, 57–60), and are therefore distinct from pelvic plexus ganglionic neurons located in mesenteries external to the bladder (17). Intramural ganglion neurons receive parasympathetic efferent inputs from pelvic splanchnic nerves (postganglionic pelvic plexus ganglion neurons, and direct preganglionic efferents from sacral cord ventral horn neurons), sympathetic efferent inputs via the hypogastric nerve (mainly from postganglionic caudal mesenteric ganglion neurons, and some direct preganglionic efferents traveling in hypogastric nerve from L1–L5 lumbar intermediolateral horn neurons), and visceral afferents (hitchhiking on thoracic, lumbar, and pelvic splanchnic nerves and vessels) (17, 22, 34). Thus, transection of sacral or L7 roots, pelvic nerves, or hypogastric nerves (each or all together) merely decentralize the bladder rather than denervate it (17, 56). We previously showed that a 3- to 6-mo sacral decentralization (transection of S1–S3 ventral and dorsal roots) resulted in no diminishment of intramural ganglia or their neurons in ventromedial regions of the midbladder compared with sham-operated control dogs (17), nor did a sacral decentralization and immediate somatic nerve transfer followed by a 4-mo recovery period (16). By 12 mo after sacral decentralization only, many healthy postganglionic intramural ganglia were found in the ventromedial bladder wall of dogs, as well as at 12 mo after a more extensive sacral root + L7 dorsal root + hypogastric nerve decentralization (17). However, the long-term decentralization used here (up to 21 mo) was more detrimental to intramural ganglia neuronal integrity (Figs. 2 and 4) than in shorter or less decentralized studies.

Bladder wall percent muscle was maintained near Sham/Unop C levels in both Decentralized and ObNT Reinn animals. We also saw a low moderate and positive correlation between ex vivo muscle strip contractility in response to KCL and detrusor muscle thickness (but not fibrosis). Thus, bladder wall percent muscle is maintained despite the long decentralization. This interpretation agrees with our prior study showing that isolated bladder smooth muscle contractility was preserved at 1 yr after similar decentralization using ex vivo testing methods (17). However, that prior study did not account for the effects of inflammation and fibrosis in the detrusor muscle layer on the recovery of bladder function. Here, as we examined for the presence of intramural ganglia only in ventromedial regions of the middle bladder in this study (in a sample of ∼2.5 mm^3^ in size), perhaps axons from neurons in intramural ganglia located in other portions of the bladder wall (dome, neck, and trigone) were still intact enough to maintain muscle mass. Future studies will examine that question. A single smooth muscle cell is known to receive input from more than one source, and smooth muscle cells are electrically coupled to each other via gap junctions (61). In addition, perhaps the increase in pgp9.5+ axon density in ObNT Reinn animals to a density comparable to Sham/Unop C animals (Fig. 5) preserved muscle integrity. In support of this latter suggestion, L1–L6 root evoked MDP in ObNT Reinn correlated positively with pgp9.5+ axon density in the detrusor muscle layer, and we have shown that transferred somatic nerves extend axons directly to detrusor smooth muscle fibers, bypassing intramural ganglia (16). With the low number of intramural ganglia and intramural ganglia neurons that could serve as the source of the increased pgp9.6+ axon density in Decentralized and ObNT Reinn animals (Fig. 4), the source of the innervation in the ObNT Reinn animals is likely in growing axons from the transferred obturator nerve. Although it has been shown that sympathetic axons can regrow or sprout from transected sacral spinal roots or hypogastric nerves after decentralization (32, 33), we observed lower numbers of tyrosine hydroxylase immunopositive axons in the bladder walls of both ObNT Reinn and Decentralized dogs in this study, indicating that sympathetic axonal regrowth was not a contributing factor.

Functional changes in ex vivo smooth muscle strip contractons reported in Part 1 of this series (18) were absent or of moderate size. In contrast, the morphological changes reported are of a more impressive magnitude. We contend that the differences may be because muscles used for the ex vivo muscle strip electrophysiological studies were denuded of mucosa, submucosa (which contains a thin longitudinal layer of smooth muscle visible in our histological images as well as a muscularis mucosae), and serosa. Also, many of the intramural ganglia that are typically located in the zone in the lower detrusor muscle layer and serosa were removed during muscle strip preparation. We examined the muscle strips microscopically after immunostaining with an axon marker (pgp9.5) and found only a few remaining intramural ganglion neurons in some of the strips, even when examining samples from control animals (data not shown). The muscle strips are also separated from their peripheral nerve and spinal cord input at the time of ex vivo testing. In contrast, results in this paper include morphological changes from intact bladders and functional changes in response to electrophysiological stimulation of spinal cord roots and extrinsic peripheral nerves innervating the bladder. Thus, contractile responses in ex vivo smooth muscle strips, while important and informative, may not exactly match those in an intact bladder with many functionally contributing layers and intrinsic and extrinsic innervation.

We have several limitations in this study. We examined only female mixed-bred mongrel hound dogs to reduce sex variability. Examination of similar nerve transfer studies in male dogs is planned for future studies. Also, this study examined only pgp9.5+ and tyrosine hydroxylase+ axons in the bladder wall. Examining other functional axonal subtypes is the topic of another study in preparation. The pilot study of this series reported on the origin of sensory axons reinnervating the bladder using retrograde dye labeling methods in a subset of these animals (14), partially reducing this limitation.

### Perspectives and Significance

In conclusion, despite the loss of intramural ganglia and ganglionic neurons, some bladder smooth muscle function was restored in reinnervated animals in which the obturator nerve, a lumbar originating somatic nerve, was transferred to the pelvic nerve’s anterior vesicle branch. We hypothesize that this was due to the change in innervation, indicated by an increase in pgp9.5+ axonal density in the ObNT Reinn animals and increased MDP in these same animals after stimulation of L1-L6 spinal roots. However, the combination of persistent inflammation in the submucosal and detrusor muscle layers, and enhanced collagen deposition in the detrusor muscle layer, occurring as a consequence of the extensive long-term decentralization, were detrimental to function. Moving forward, we propose pursuing surgical reinnervation closer to the time of injury or provision of anti-inflammatory or antifibrotic drugs to avoid the negative consequences of inflammation and fibrosis on healing.

## GRANTS

Research reported in this publication was supported by the National Institute of Neurological Disorders and Stroke of the National Institutes of Health under Award Number R01NS070267 (to M.R.R. and M.F.B.). The high definition video cystoscopy system used for the retrograde dye injections was supplied from a Customer Initiated Equipment Grant from Karl Storz Endoscopy-America, Inc., El Segundo, CA.

## DISCLAIMERS

The content is solely the responsibility of the authors and does not necessarily represent the official views of the National Institutes of Health.

## DISCLOSURES

Michael R. Ruggieri, Sr. received the Video Cystoscopy Equipment Grant from KarlStorz America. None of the other authors has any conflicts of interest, financial or otherwise, to disclose.

## AUTHOR CONTRIBUTIONS

M.F.B., C.L.T., A.S.B., E.P.D., J.M.B., M.M., and M.R.R. conceived and designed research; M.F.B., C.L.T., G.E.C., N.A.F., E.T., L.J.H., B.S.M., D.S.P., D.G., A.S.B., E.P.D., M.A., J.M.B., M.M., M.A.P., I.J.W., and M.R.R. performed experiments; M.F.B., C.L.T., G.E.C., N.F., E.T., L.J.H., D.S.P., D.G., A.S.B., E.P.D., M.A., and M.R.R. analyzed data; M.F.B., C.L.T., G.E.C., D.S.P., D.G., A.S.B., E.P.D., J.M.B., M.M., M.A.P., I.J.W., and M.R.R. interpreted results of experiments; M.F.B. and C.L.T. prepared figures; M.F.B. and C.L.T. drafted manuscript; M.F.B., C.L.T., M.A.P., I.J.W., and M.R.R. edited and revised manuscript; M.F.B., C.L.T., G.E.C., N.A.F., E.T., L.J.H., B.S.M., D.S.P., D.G., A.S.B., E.P.D., M.A., J.M.B., M.M., M.A.P., I.J.W., and M.R.R. approved final version of manuscript.
